# Epidemiological survey of calf diarrhea related viruses in several areas of Guangdong Province

**DOI:** 10.3389/fmicb.2024.1441419

**Published:** 2024-09-16

**Authors:** Jinping Chen, Wenxin Meng, Huijuan Zeng, Jingyu Wang, Shizhe Liu, Qifeng Jiang, Zihan Chen, Zihang Ma, Zhen Wang, Shoujun Li, Kun Jia

**Affiliations:** ^1^College of Veterinary Medicine, South China Agricultural University, Guangzhou, China; ^2^Guangdong Technological Engineering Research Center for Pet, Guangzhou, China

**Keywords:** calf diarrhea, viruses, epidemic situation, genetic evolution analysis, Guangdong Province

## Abstract

**Introduction:**

Bovine torovirus (BToV), Bovine enterovirus (BEV), Bovine norovirus (BNoV), Bovine coronavirus (BCoV), Bovine rotavirus (BRV), and Bovine viral diarrhea virus (BVDV) are significant pathogens causing diarrhea in calves, characterized by their high prevalence and challenging prevention and control measures.

**Methods:**

We analyzed 295 calf diarrhea samples, amplifying the *M* gene from BToV-positive samples, the *5’UTR* gene from BEV-positive samples, the *RdRp* gene from BNoV-positive samples, the *VP7* gene from BRV-positive samples, the *S* gene from BCoV-positive samples, and the *5’UTR* gene from BVDV-positive samples. Subsequent homology analysis and phylogenetic tree construction were performed.

**Results:**

The overall viral positive rate in Guangdong Province was 21.36%. Specific detection rates were as follows: Foshan City at 50.00% (18/36), Guangzhou City at 43.90% (36/82), Huizhou City at 21.21% (7/33), Yangjiang City at 2.08% (1/48), Meizhou City at 1.39% (1/72), and Heyuan City at 0.00% (0/24). The detection rates for BToV, BEV, BNoV, BCoV, BRV, and BVDV were 0.34% (1/295), 6.10% (18/295), 0.68% (2/295), 1.36% (4/295), 10.85% (32/295), and 2.03% (6/295), respectively. Notably, the highest overall virus detection rate was observed in the Guangzhou-Foshan region, with BRV and BEV showing the highest detection rates among the six viruses. This study marks the first report of BToV and BNoV in Guangdong Province. Phylogenetic analysis revealed that the BToV strain belonged to type II, sharing genetic similarities with epidemic strains from various provinces in China. The BEV strains were categorized into E and F types, with the F type being the predominant strain in Guangdong Province and exhibiting the closest genetic relationship to strains from Heilongjiang and Guangxi. The BNoV strains, along with Hebei strains, were identified as GIII.2 subgenotype. BCoV strains showed the highest genetic similarity to strains from Sichuan. All BRV strains were classified under the G6 subtype and had the closest genetic relationship with human rotavirus strains. BVDV strains were identified as subtype 1b, closely related to the Beijing strain. In conclusion, this study investigated the prevalence and evolutionary characteristics of diarrhea-associated viruses in calves in specific areas of Guangdong Province, providing a valuable reference for establishing effective prevention and control measures in cattle farms.

## 1 Introduction

Calf diarrhea is a significant factor impacting the development of the cattle industry. The direct and indirect economic losses due to calf diarrhea can reach 10 billions of yuan annually, undermining the confidence of farmers and breeding enterprises and severely hindering the industry’s growth. Apart from foodborne factors, the causes of mass calf diarrhea can be broadly categorized into three categories: bacterial, viral, and parasitic. Reports indicate that the incidence of viral-induced diarrhea in calves is on the rise annually, underscoring the importance of focusing on this issue (Hou et al., 2019; Lee et al., 2019). The following is a concise summary of the viruses responsible for causing diarrhea in calves.

Bovine torovirus (BToV) is a single-stranded positive-sense RNA virus belonging to the family *Toroviridae*, *order Nidovirales* (Hoet and Saif, 2004; Hu et al., 2019). Bovine enterovirus (BEV) is a single-stranded RNA virus without an envelope, belonging to the family *Picornaviridae*, *genus Enterovirus* (Beato et al., 2018). Bovine norovirus (BNoV) is a non-enveloped RNA virus belonging to the family *Caliciviridae, genus Norovirus* (Hardy, 2005). Bovine coronavirus (BCoV) is a single-stranded RNA virus belonging to the family *Coronaviridae, order Nidovirales.* Bovine rotavirus (BRV) belongs to the family *Reoviridae, genus Rotavirus*, and is a double-stranded RNA virus. Bovine viral diarrhea virus (BVDV) is a RNA virus belonging to the genus *Pestivirus*, family *Flaviviridae*. All six viruses can infect calves, causing symptoms such as loss of appetite, diarrhea, and dehydration, which seriously affect the growth and development of calves and can even lead to death in severe cases (Su et al., 2021; Toftaker et al., 2017). Detection of these viruses has been reported in many countries worldwide (Ito et al., 2007; Kirisawa et al., 2007; Lojkić et al., 2015; Yeşilbağ et al., 2017; Zhu et al., 2022).

In the early stages, our laboratory collected 295 fecal and anal swabs from calves with diarrhea on large-scale cattle farms in Guangzhou, Foshan, Huizhou, Yangjiang, Jiangmen, and Heyuan of Guangdong Province from 2021 to 2023 for the detection of the aforementioned six viruses (Meng et al., 2023). Subsequently, the positive samples underwent homology analysis, and a phylogenetic tree was constructed. This analysis is crucial for understanding and elucidating the genetic variation of diarrhea-related viruses in calves in Guangdong Province. It lays the foundation for molecular epidemiological studies of these viruses, providing valuable insights for prevention, control, and surveillance efforts.

## 2 Materials and methods

### 2.1 Sample collection

**Figure 1:**
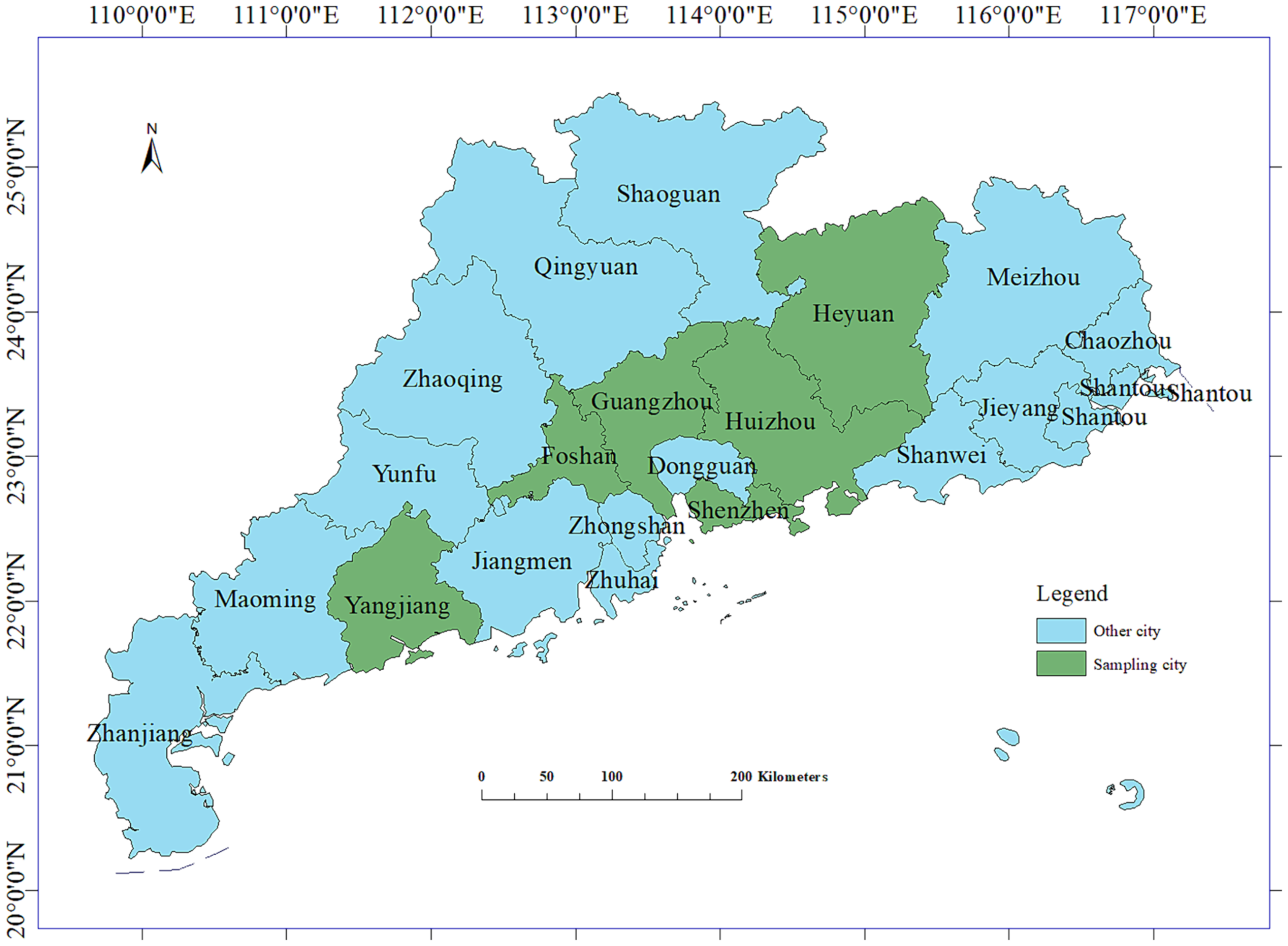
Longitude and latitude map of the sampling areas.

Between 2021 and 2023, a total of 295 fecal and anal swabs were collected from calves with diarrhea in the Guangzhou, Foshan, Huizhou, Yangjiang, Jiangmen, and Heyuan areas of Guangdong Province (Figure 1) for the detection of diarrhea-associated viruses. The specific data are presented in Table 1.

**Table 1:** Source of sample information.

Region	Guangzhou	Foshan	Meizhou	Huizhou	Yangjiang	Heyuan	Total
Sample size	82	36	72	33	48	24	295

### 2.2 Main reagents

Total RNA rapid extraction kit (Shanghai Feijie Biotechnology Co., Ltd.), high-purity low electrotonic agarose (Qingke Biotechnology Co., Ltd.), 2× Taq PCR Star Mix (Beijing Kangruncheng Biotechnology Co., Ltd.), 2000DL DNA Marker (Beijing Jinsha Biotechnology Co., Ltd.), HiScript II 1st Strand cDNA Synthesis Kit (Nanjing Nuoweizan Biotechnology Co. Ltd.), XKL-GelRed nucleic acid dye (Guangzhou Xinkelai Biotechnology Co., Ltd.).

### 2.3 RNA extraction and cDNA synthesis

Sample RNA was extracted using the RNAfast200 kit (Shanghai Feijie Bio-Technology Co., Ltd., Shanghai, China). PBS solution (1 mL) was added to 10 mg feces or anal swabs and mixed thoroughly. Samples were then centrifuged at 12000 rpm at 4°C for l min. The supernatant was removed and set aside. Lysate (500 μL) was added, the mixture placed upside down, and allowed to stand for l min. The lysed samples were transferred to an inner cannula and centrifuged at 12000 rpm for l min at 4°C. The liquid was discarded, wash solution (500 μL) added to the inner cannula, the mixture was centrifuged for l min at 12000 rpm and 4°C, the liquid discarded, and the procedure was repeated. After the final liquid discarding step, the samples were centrifuged at 12000 rpm for l min at 4°C. The inner cannula was transferred to a clean EP tube and allowed to sit for 3–5 min. After the liquid was completely evaporated, 25–50 μL elution buffer was added to the center of the membrane, left at room temperature for l min, centrifuged at 12000 rpm at 4°C for 1 min, and sample RNA was obtained. RNA quality was assessed using a Nano Drop One ultramicrospectrophotometer (Thermo Fisher Scientifc., Shanghai, China), and stored at-80°C until further use. RNA was reverse-transcribed to cDNA using HiScript III All-in-one RT SuperMix (Nanjing Vazyme Biotech Co., Ltd., Nanjing, China) and stored at −20°C until use. Utilizing a multiplex real-time fluorescent quantitative PCR assay (Meng et al., 2023), we screened 295 samples for viral presence. Subsequently, the positive samples identified underwent specific amplification of the target gene.

### 2.4 Target gene amplification

The *M* gene of BToV-positive samples, the *5’UTR* sequence of BEV-positive samples, the *Rdrp* gene of BNoV-positive samples, the *VP7* gene of BRV-positive samples, the *S* gene of BCoV-positive samples, and the *5’UTR* sequences of BVDV-positive samples were amplified by PCR. The primer sequences are shown in Table 2. The reaction system comprised 10 μL of 2 × Taq PCR Star Mix, 1 μL each of the upper and downstream primers, 2 μL of cDNA template, and dd water supplemented to 20 μL. The reaction procedure included an initial denaturation at 95°C for 5 min; 35 cycles of 95°C for 30 s, 50–60°C for 30 s, and 72°C for 1 min; The PCR products were examined by agarose gel electrophoresis at 1.5% concentration at 72°C for 5 min.

**Table 2:** Target gene amplification PCR primer sequence.

Primer reference sequence accession no.	Primer	Sequence (5′-3′)	Size
MN882587.1	BToV-*M*-F	TGAGATGAATGGCGAGGTTA	901 bp
	BToV-*M*-R	GCAGTCAGACCTACTATCACCA	
MK639928.1	BEV-*5’UTR*-F	GAACCTTTGTACGCCTGTT	487 bp
	BEV-*5’UTR*-R	GGATTAGCAGCATTCACG	
MN480761.1	BNoV-*RdRp*-F	CCCTCCCTACAACAGGTCAT	1,294 bp
	BNoV-*RdRp*-R	GAAATCTCATCCAAGCAAACAT	
JN831208.1	BRV-*VP7*-F	ATGTATGGTATTGAATATACCACA	863 bp
	BRV-*VP7*-R	ACTTGCCACCATTTTTTC	
ON142320.1	BCoV-*S*-F	CGATCAGTCCGACCAATCTA	1,150 bp
	BCoV-*S*-R	GAGGTAGGGGTTCTGTTGCC	
MN417910.1	BVDV-*5’UTR*-F	CCATGCCCTTAGTAGGACTAGC	280 bp
	BVDV-*5’UTR*-R	GTGCCATGTACAGCAGAGATT	

### 2.5 Genome sequencing

The amplified DNA fragments were excised and submitted to Shanghai Sangon Biological Co., Ltd. for sequencing analysis.

### 2.6 Homology analysis and phylogenetic tree construction

The sequencing results were compared with each virus sequence registered in NCBI. The *M* gene sequence of the representative BToV reference strain, the *5’UTR* sequence of the BEV reference strain, the *Rdrp* gene sequence of the BNoV reference strain, the *VP7* gene sequence of the BRV reference strain, the *S* gene sequence of the BCoV reference strain, and the *5’UTR* sequence of the BVDV reference strain were selected. Bioinformatics software Megalign and MEGA 11 were used for homology analysis and phylogenetic tree construction.

## 3 Results

### 3.1 PCR detection results of each virus

The data obtained from the viral detection in 295 samples via multiplex real-time PCR are delineated in Table 3.

**Table 3:** PCR detection results of various viruses.

Region	Sample size	The number of positive detection of each virus						Positive rate
		BToV	BEV	BNoV	BCoV	BRV	BVDV	
Guangzhou	82	1	1	0	3	27	4	36/82 (43.90%)
Foshan	36	0	12	1	0	5	0	18/36 (50.00%)
Meizhou	72	0	1	0	0	0	0	1/72 (1.39%)
Huizhou	33	0	4	1	1	0	1	7/33 (21.21%)
Yangjiang	48	0	0	0	0	0	1	1/48 (2.08%)
Heyuan	24	0	0	0	0	0	0	0/24 (0.00%)
Total	295	1 (0.34%)	18 (6.10%)	2 (0.68%)	4 (1.36%)	32 (10.85%)	6 (2.03%)	63/295 (21.36%)

### 3.2 Homology and genetic evolution analysis of BToV *M* gene

**Figure 2:**
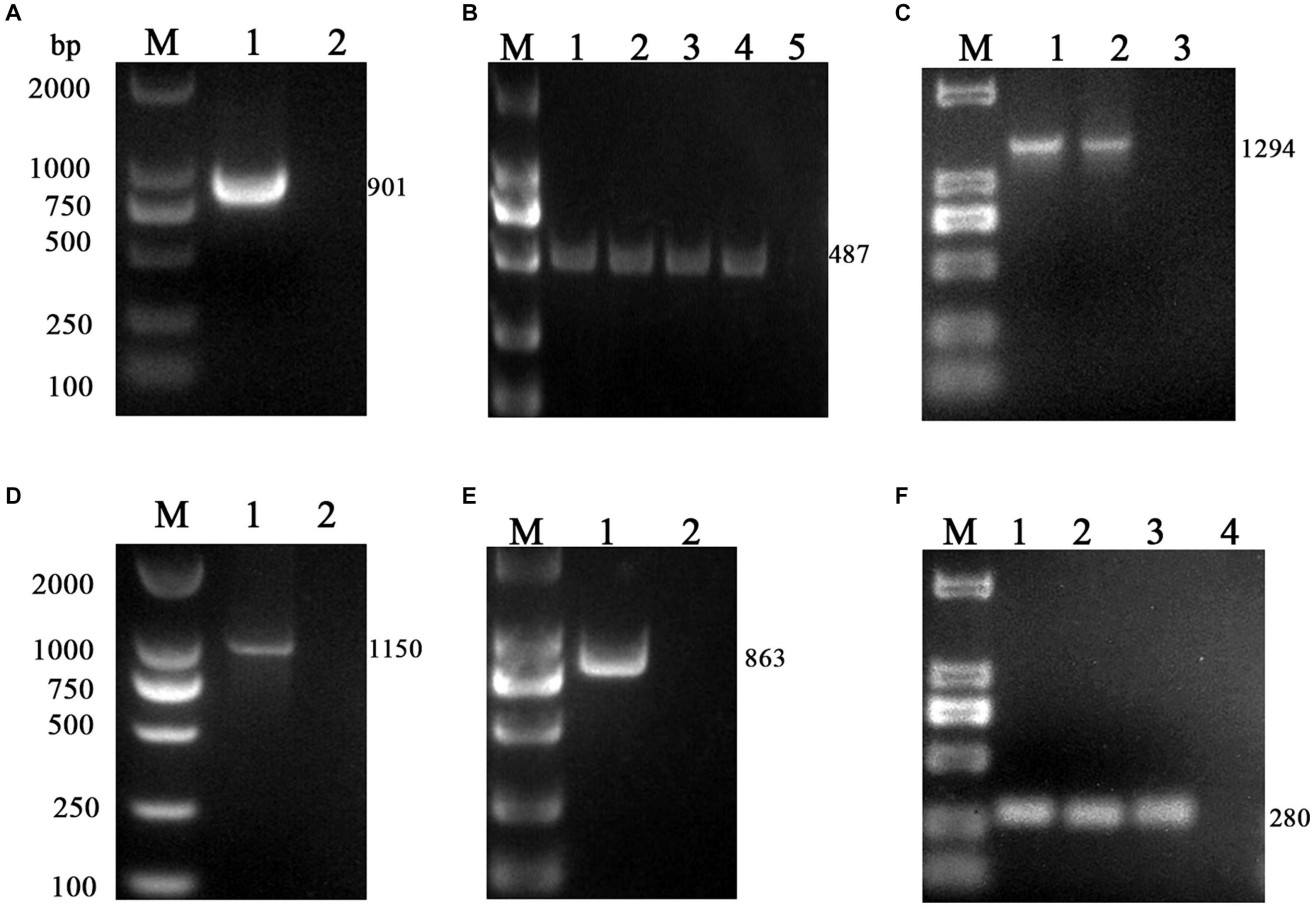
PCR results of six virus-positive samples. **(A)** BToV: M, DL2000 DNA Marker; 1, Amplification of *M* gene from BToV-positive samples in Guangzhou showed a fragment size of 901 bp; 2, negative control. **(B)** BEV: M, DL2000 DNA Marker; 1, 2, 3, 4 Amplification of the *5’UTR* gene from BEV-positive samples in Guangzhou/Foshan/Meizhou/Huizhou showed a fragment size of 487 bp; 5 negative control. **(C)** BNoV: M, DL2000 DNA Marker; 1, 2 Amplification of the *Rdrp* gene from BNoV-positive samples in Foshan/Huizhou showed a fragment size of 1,294 bp; 3, negative control. **(D)** BCoV: M, DL2000 DNA Marker; 1 Amplification of the *S* gene from BCoV-positive samples in Guangzhou showed a fragment size of 1,150 bp; 2 negative control. **(E)** BRV: M, DL2000 DNA Marker; 1 Amplification of the *VP7* gene from BRV-positive samples in Foshan showed a fragment size of 863 bp; 2 negative control. **(F)** BVDV: M, DL2000 DNA Marker; 1, 2, 3 Amplification of the *5’UTR* gene from BVDV-positive samples in Guangzhou/Huizhou/Yangjiang showed a fragment size of 280 bp; 4 negative control.

The *M* gene of the BToV-positive samples was amplified using the primers in Table 2, one target fragment was amplified, and the electrophoresis results are depicted in Figure 2. The amplified band was approximately 901 bp, consistent with the size of the target fragment.

**Figure 3:**
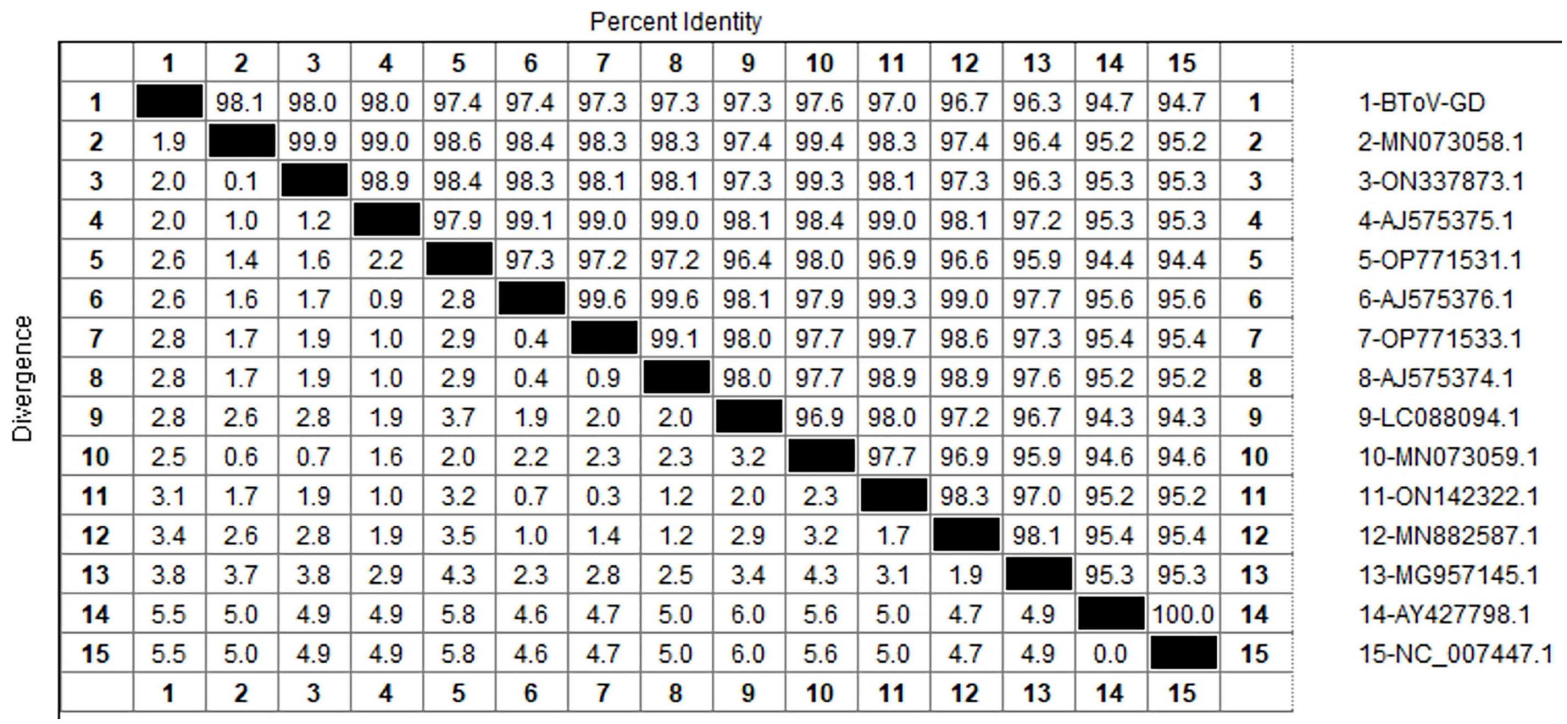
Homology analysis of BToV *M* gene.

After sequencing the amplified bands, homology analysis of the BToV *M* gene sequence was performed using Megalign software, and the results are presented in Figure 3. The homology of the *M* gene between Guangdong BToV strains and other strains was 94.7–98.1%, with the homology between Guangdong BToV strains and representative strains in China (MN073058.1, ON337873.1, AJ575375.1) ranging from 98.0 to 98.1%. The lowest homology was 94.7% with the classical strain “Breda” (NC007447.1). Compared with the classical strain “Breda” (NC007447.1), there were 37 base mutations and nine amino acid changes in the *M* gene sequence of the Guangdong BToV strain. Compared with the representative strain in China (MN073058.1), there were only 10 base mutations in the *M* gene sequence of the Guangdong BToV strain.

**Figure 4:**
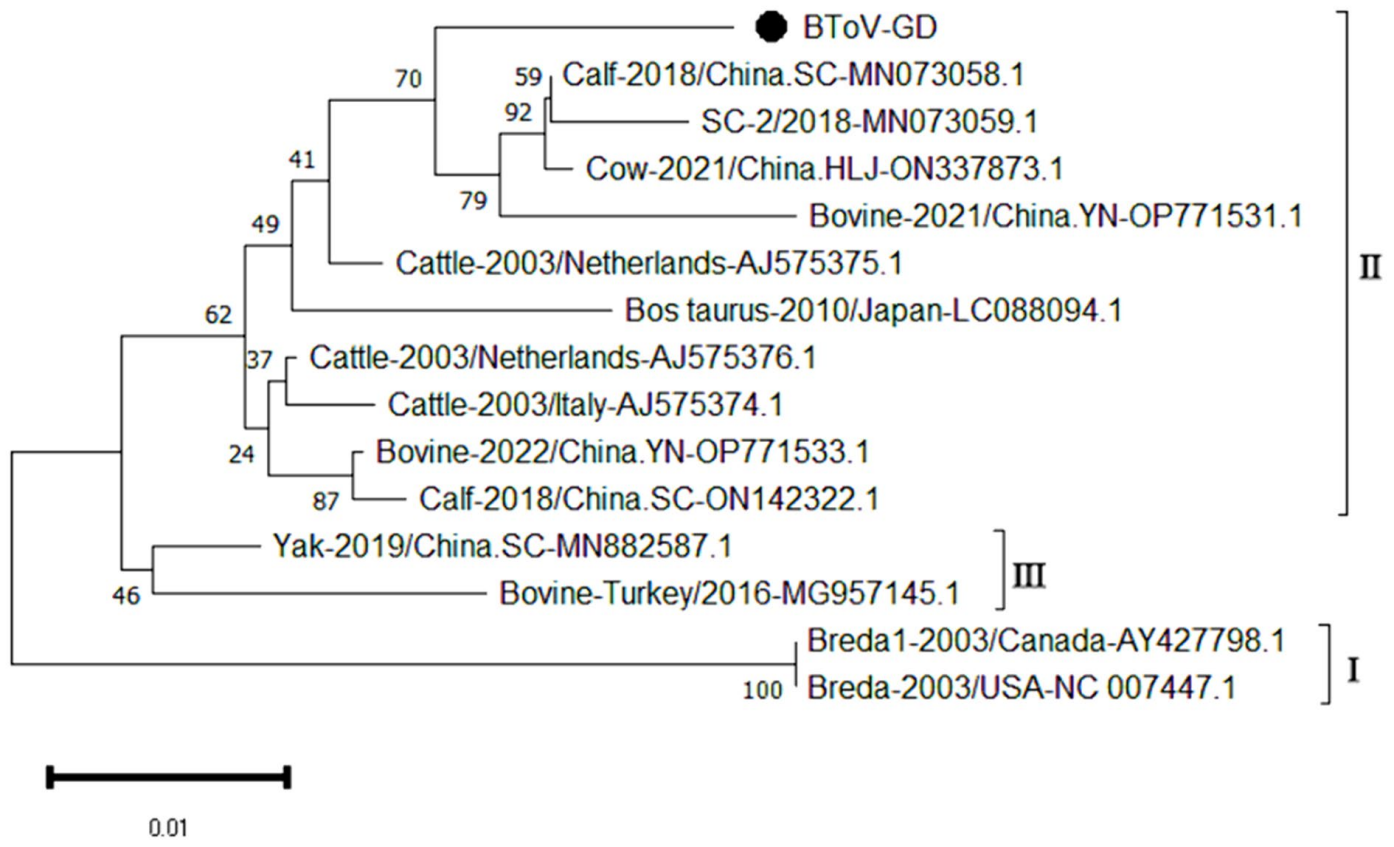
Phylogenetic tree based on the *M* gene nucleotides of BToV. The sequences of the resulting BToV-positive samples have been marked with a black dot before the sequence name, each sequence is named “species-year/region-accession number,” and the virus typing is indicated in curly braces on the right.

The genetic evolution tree was constructed using MEGA 11, and the results are illustrated in Figure 4. The phylogenetic analysis revealed that the Guangdong BToV strain belongs to type II, clustering within the same branch as strains from other provinces in China. In contrast, it shows a distant relationship with the classical “Breda” strain.

### 3.3 Homology and genetic evolution analysis of BEV *5’UTR* gene

Using the primers listed in Table 2, the *5’UTR* sequences of BEV-positive samples were amplified, one target fragment was amplified, the electrophoresis results are shown in Figure 2. The amplified band was approximately 487 bp, which is consistent with the expected size of the target fragment.

**Figure 5:**
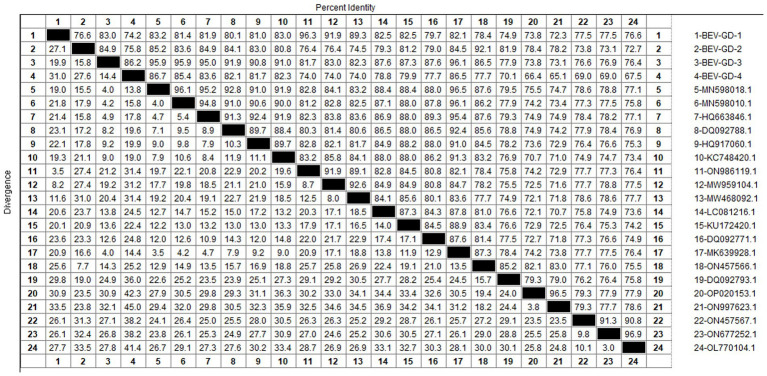
Homology analysis of BEV *5’UTR* gene.

After sequencing the amplified bands, homology analysis of BEV *5*’*UTR* sequences was performed using Megalign software, and the results are shown in Figure 5. Both genotype E and F of BEV strains coexist in Guangdong. The overall homology of the *5’UTR* region among BEV strains in Guangdong is 65.1–98.3%, with genotype E strains showing a homology of 75.5–96.9%, and genotype F strains exhibiting a homology of 81.2–96.5%. The gene sequence homology among Guangdong BEV strains ranges from 73.6 to 98.3%. Compared with the Jilin BEV strain (MN598018.1), the *5’UTR* sequence of the Guangdong BEV-GD-4 strain underwent 49 nucleotide substitutions, 4 nucleotide insertions, and resulted in 23 amino acid changes. The *5’UTR* sequence of the BEV-GD-3 strain experienced 15 nucleotide mutations, 2 nucleotide deletions, and 8 amino acid alterations.

**Figure 6:**
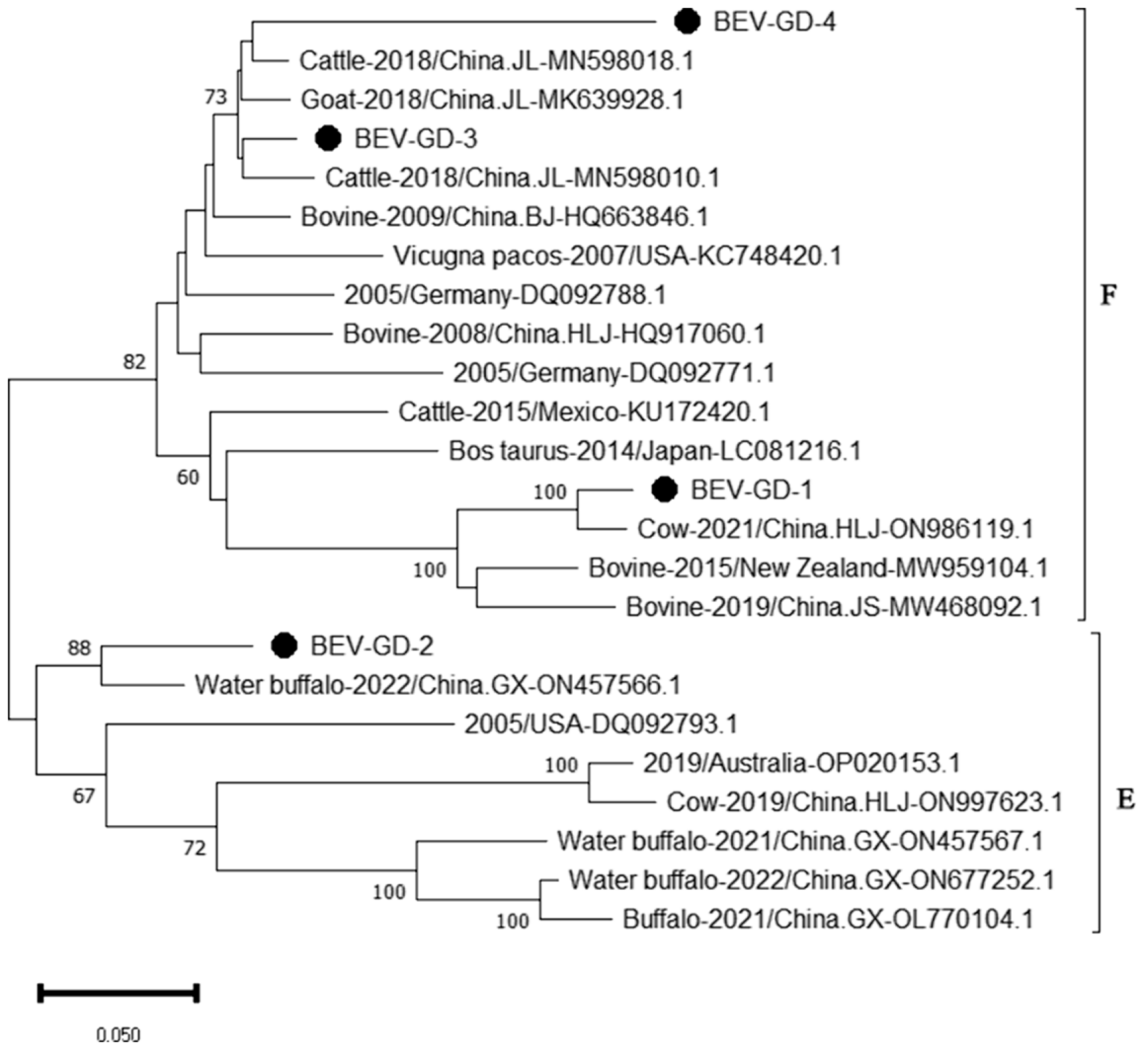
Phylogenetic tree based on the *5’UTR* gene nucleotides of BEV. The sequences of the resulting BEV-positive samples have been marked with a black dot before the sequence name, each sequence is named “species-year/region-accession number,” and the virus typing is indicated in curly braces on the right.

The genetic evolution tree was constructed using MEGA 11, as shown in Figure 6. The phylogenetic tree indicates that BEV isolated in Guangdong province could be divided into three branches. Some of these strains were in the same branch as the strains isolated in Henan (MN598018) and Shandong (MK639928). Another group of strains was in the same branch as the Heilongjiang strain (ON986119), belonging to type F. A separate strain was in the same branch as the strain from Guangxi buffalo (ON457566), belonging to genotype E.

### 3.4 Homology and genetic evolution analysis of BNoV *Rdrp* gene

Using the primers in Table 2, the *Rdrp* gene of the BNoV positive samples was amplified, one target fragment was amplified, and the electrophoresis results are shown in Figure 2. The amplified band was approximately 1,294 bp, which was consistent with the size of the target fragment.

**Figure 7:**
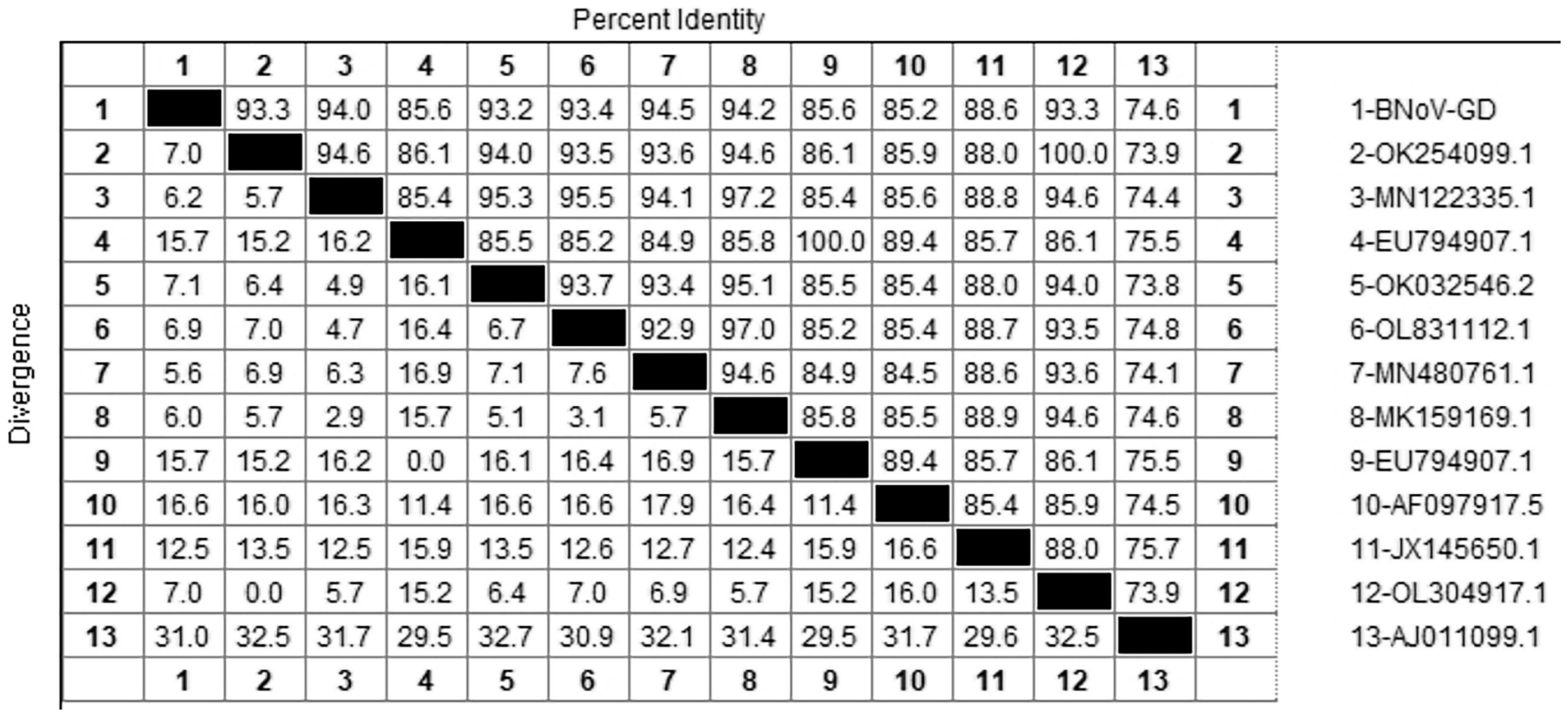
Homology analysis of BNoV *Rdrp* gene.

**Figure 8:**
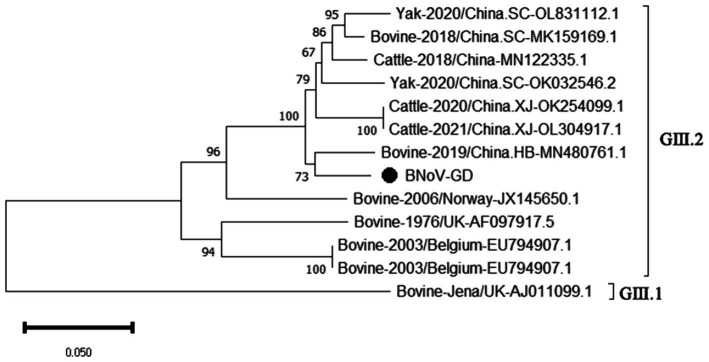
Phylogenetic tree based on the *Rdrp* gene nucleotides of BNoV. The sequences of the resulting BNoV-positive samples have been marked with a black dot before the sequence name, each sequence is named “species-year/region-accession number,” and the virus typing is indicated in curly braces on the right.

After sequencing the amplified bands, the BNoV *Rdrp* gene sequence was analyzed for homology using Megalign software, and the results are shown in Figure 7. The nucleotide sequence homology of the *RdRp* gene of BNoV in this study ranged from 74.6 to 94.5% when compared to representative strains. The lowest homology, 74.6%, was observed with the classic strain “Jena” (AJ011099.1). In contrast, the homology with international strains was slightly higher, ranging from 85.2 to 88.6%. For domestic strains from other provinces in China, the homology was higher, ranging from 93.2 to 94.5%, with the highest similarity of 94.5% observed with a strain from Hebei (MN480761.1). When compared to the classical “Jena” strain (AJ011099.1), the *RdRp* gene of the Guangdong BNoV strain exhibited 254 base substitutions, two deletions, and 43 amino acid alterations. The genetic evolution tree was constructed using MEGA 11, as shown in Figure 8 The phylogenetic tree showed that the BNoV strain from Guangdong was in the same branch as strains from several other provinces in China, belonging to the genotype GIII.2. It was closest to the Hebei strain (MN480761.1), which was in the same branch.

### 3.5 Homology and genetic evolution analysis of BRV *VP7* gene

Using the primers listed in Table 2, the *VP7* gene of the BRV-positive samples was amplified, one target fragment was amplified. The electrophoresis results are shown in Figure 2. The amplified band was approximately 863 bp, consistent with the expected size of the target fragment.

**Figure 9:**
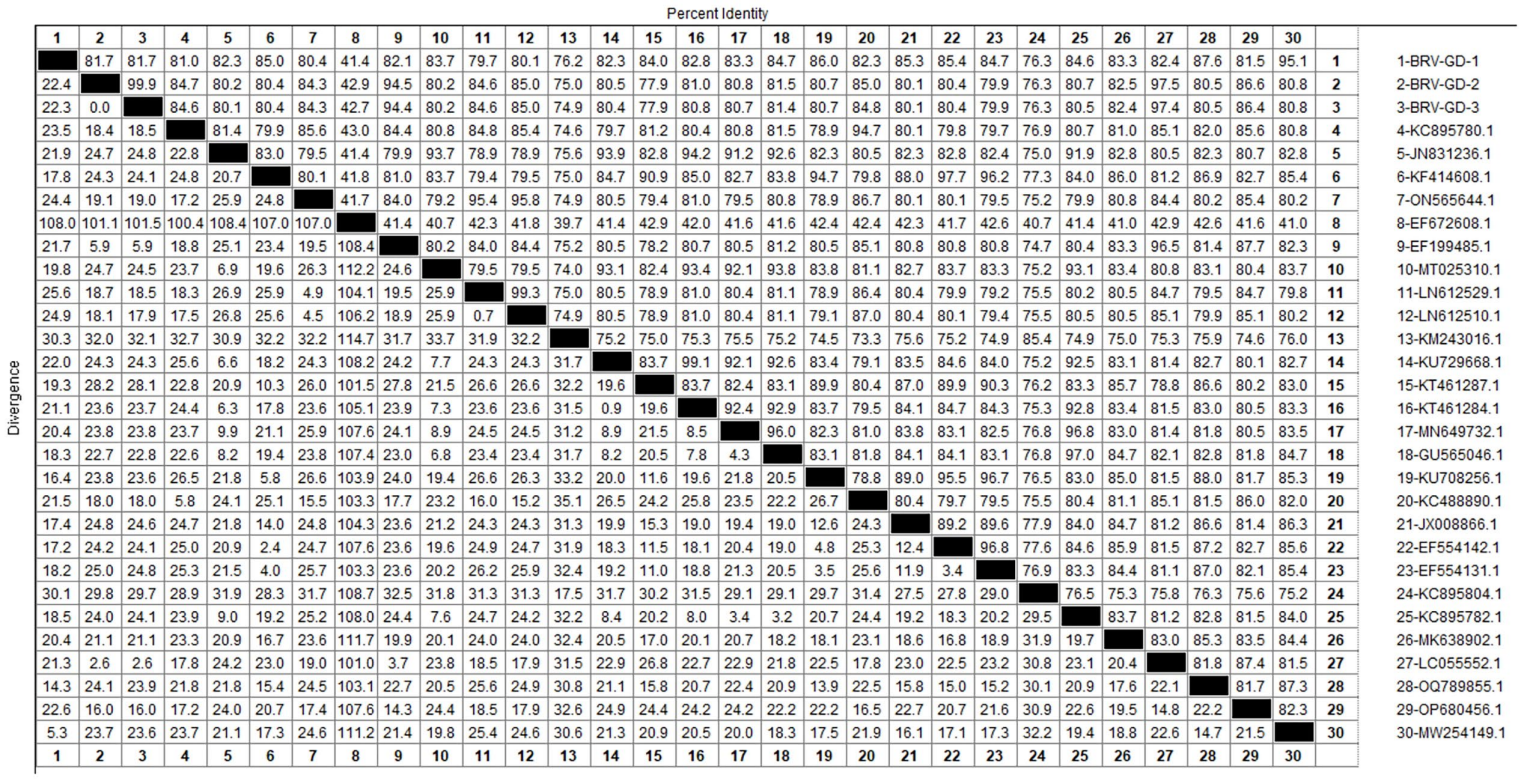
Homology analysis of BRV *VP7* gene.

After sequencing the amplified bands, homology analysis of the BRV *VP7* gene sequence was performed using Megalign software, and the results are shown in Figure 9. The homology of the *VP7* gene of BRV with representative rotavirus strains ranged from 41.4 to 95.1%. The lowest homology was 41.1% with the human rotavirus strain (EF672608.1) from the United States. The highest homology (94.4–97.5%) was with rotavirus strains isolated from cattle in India (EF199485.1), humans in Thailand (LC055552.1), and human sewage in Shandong province (MW254149.1). Compared with the human-derived rotavirus strain from Shandong (MW254149.1), the *VP7* gene sequence of the Guangdong BRV-GD-1 strain underwent 34 nucleotide mutations, resulting in six amino acid changes. The *VP7* gene sequence of the Guangdong BRV-GD-2 strain, on the other hand, experienced 107 nucleotide mutations, leading to 22 amino acid alterations.

**Figure 10:**
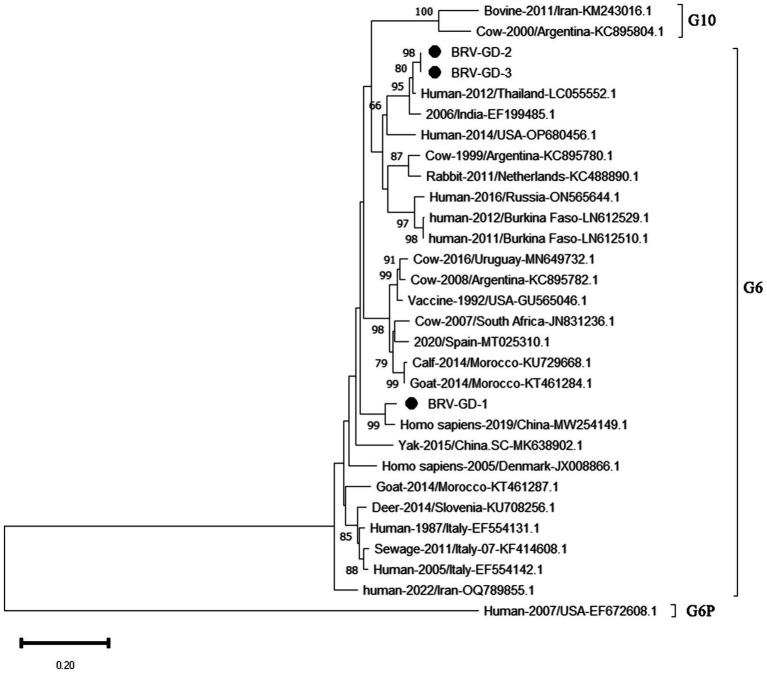
Phylogenetic tree based on the *VP7* gene nucleotides of BRV. The sequences of the resulting BRV-positive samples have been marked with a black dot before the sequence name, each sequence is named “species-year/region-accession number,” and the virus typing is indicated in curly braces on the right.

The genetic evolution tree was constructed using MEGA 11, as shown in Figure 10. The phylogenetic tree showed that all strains belonged to genotype G6. One strain was closely related to human rotavirus (LC055552.1) and bovine rotavirus (EF199485.1), while another strain was closely related to human sewage rotavirus (MW254149.1) and was in the same branch.

### 3.6 Homology and genetic evolution analysis of BCoV *S* gene

Using the primers listed in Table 2, the *S* gene of the BCoV-positive samples was amplified, one target fragment was amplified. The electrophoresis results are shown Figure 2. The amplified band was approximately 1,150 bp, consistent with the expected size of the target fragment.

**Figure 11:**
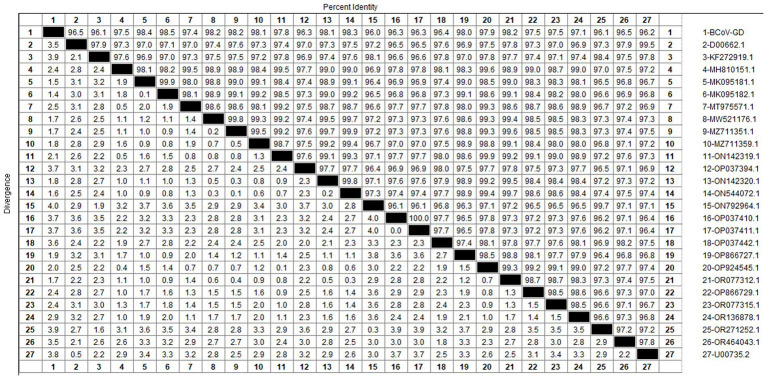
Homology analysis of BCoV *S* gene.

After sequencing the amplified bands, homology analysis of the BCoV *S* gene sequence was performed using Megalign software, and the results are shown in Figure 11. The homology of the *S* gene of Guangdong BCoV strains with representative strains ranged from 96.1 to 98.5%. The lowest homology was with the classical strains “Mebus” (U00735.2) and “Quebec” (D00662.1), at 96.1–96.2%. The highest homology, 95.4–98.5%, was with Sichuan strains (MK095181.1, MK095182.1). Compared with the classic strain “Mebus” (U00735.2), the *S* gene sequence of the Guangdong BCoV strain underwent 36 nucleotide mutations and 1 deletion.

**Figure 12:**
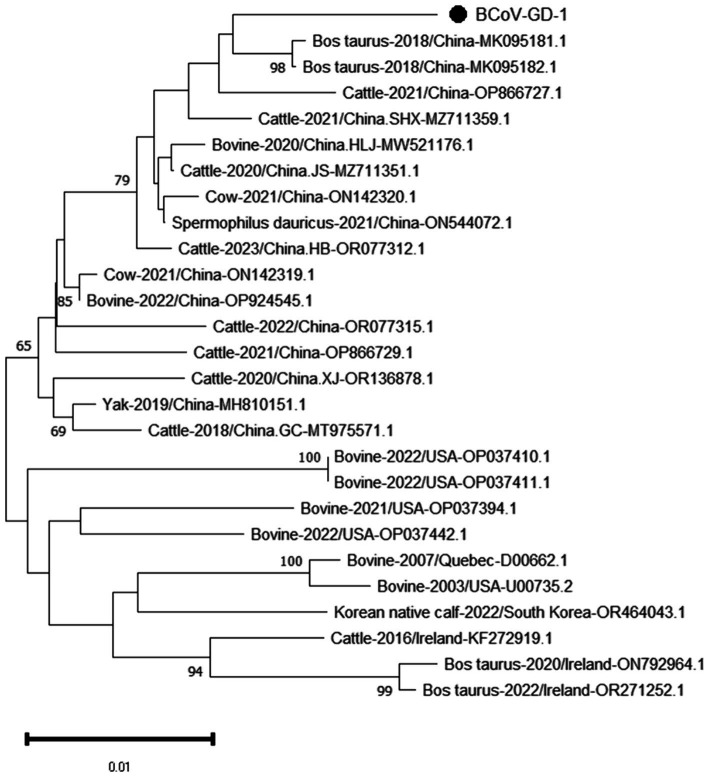
Phylogenetic tree based on the *S* gene nucleotides of BCoV. The sequences of the resulting BCoV-positive samples have been marked with a black dot before the sequence name, each sequence is named “species-year/region-accession number,” and the virus typing is indicated in curly braces on the right.

The genetic evolution tree was constructed using MEGA 11, as shown in Figure 12. The *S* gene of one BCoV strain was successfully amplified, and the phylogenetic tree showed that this strain was in the same small branch as the Sichuan strains (MK095181.1 and MK095182.1), and in the same large branch as the Shaanxi strains (OP866727.1) and Shandong strains (MZ711359.1). The domestic strains were clustered in the same branch, while the classical strains “Mebus” and “Quebec” were in a different branch with other foreign strains, indicating a distant genetic relationship.

### 3.7 Homology and genetic evolution analysis of BVDV *5’UTR* sequences

Using the primers in Table 2, the *5’UTR* sequences of BVDV-positive samples were amplified, and the electrophoresis results are shown in Figure 2. The amplified band was approximately 280 bp, which was consistent with the size of the target fragment.

**Figure 13:**
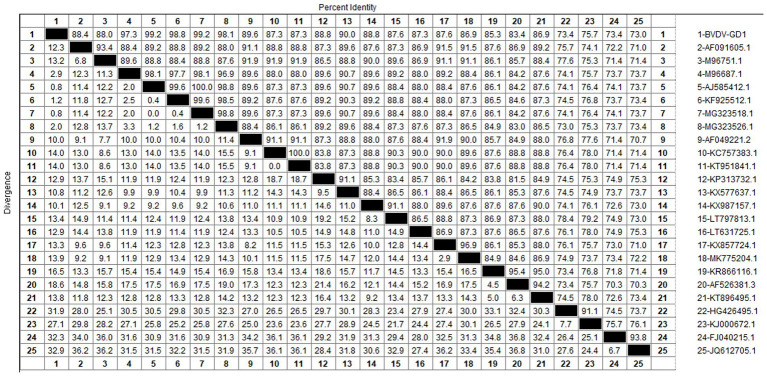
Homology analysis of BVDV *5’UTR* gene.

After sequencing the amplified bands, homology analysis of the BVDV *5’UTR* sequence was performed using Mealign software, and the results are shown in Figure 13. The homology of *5’UTR* sequences of Guangdong BVDV strains with representative strains was 73.0–99.6%. Specifically, the homology with representative strains of BVDV 1 was 83.4–99.6%, with BVDV 2 strains was 73.4–76.4%, and with BVDV 3 strains was 73.0–74.1%. The *5’UTR* sequence of the Guangdong BVDV strain showed no alterations when compared with the type 1 representative strain (MG323518.1), indicating that these two virus strains might possibly be derived from the same geographic region.

**Figure 14:**
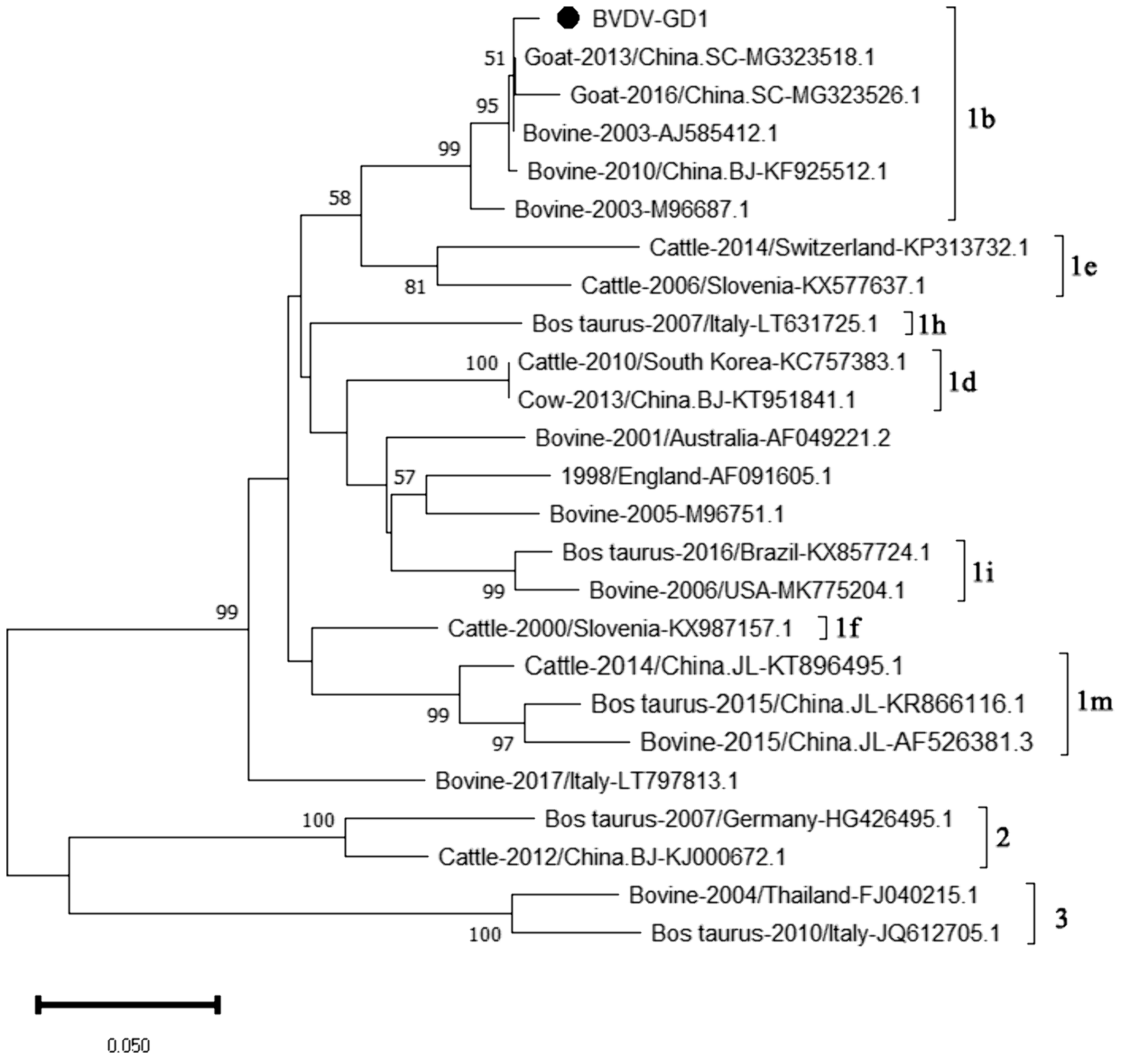
Phylogenetic tree based on the *5’UTR* gene nucleotides of BVDV. The sequences of the resulting BVDV-positive samples have been marked with a black dot before the sequence name, each sequence is named “species-year/region-accession number,” and the virus typing is indicated in curly braces on the right.

The genetic evolution tree was constructed using MEGA 11, as shown in Figure 14. The phylogenetic tree showed that the Guangdong strain and the Beijing strain (KF925512.1) were in the same branch and belonged to the BVDV-1b subtype.

## 4 Discussion

Among the 295 samples of calf diarrhea collected from multiple cities in Guangdong Province from 2021 to 2023, the overall detection rate of viruses was 21.36%. The detection rates varied significantly across different regions. Foshan had the highest detection rate at 50.00%, followed by Guangzhou at 43.90%, Huizhou at 21.21%, Yangjiang at 2.08%, and Meizhou at 1.39%. Heyuan had the lowest detection rate, with no viruses detected (0%). The higher detection rates in Foshan and Guangzhou may be attributed to frequent trade, high transportation frequency of personnel and materials, increased risk of virus transmission, and greater challenges in prevention and control in these developed areas.

Six types of viruses, namely BToV, BEV, BNoV, BCoV, BRV, and BVDV, were detected in Guangdong Province. The highest detection rates were for BRV (10.85%) and BEV (6.10%). This was followed by BVDV (2.03%), BCoV (1.36%), BNoV (0.68%), and BToV (0.34%).

The study reported that the detection rate of BToV across China was as follows: Xizang (11.76%) (Zhao et al., 2021), Henan (3.62%) (Shi et al., 2020), northeast China (4.64%) (Zhu et al., 2024) and Ningxia (4.09%) (Wang et al., 2023). Our analysis reveals that the detection rate of Bovine Torovirus (BToV) in Guangdong province was 0.34%, a figure that is notably lower compared to the rates observed in the aforementioned provinces. Based on the characteristics of the BToV gene sequence, BToV can be classified into three genotypes (I ~ III), with types II and III being the most prevalent worldwide. Genetic evolution analysis of the BToV *M* gene revealed that the Guangdong strain and strains from other provinces in China belong to genotype II. Additionally, the Guangdong strain and the classical “Breda” strain are not in the same branch, indicating a distant genetic relationship. Domestic type II strains clustered into two distinct clades, with the closest genetic affinity to strains from the Netherlands and Japan, respectively, demonstrating the genetic diversity of type II strains within the country. Since its first report in 1982 (Woode et al., 1982), there has been a lack of systematic epidemiological data on BToV in China. This study supplements the prevalence of the virus within Guangdong Province.

The study reported that the detection rate of BEV across China was as follows: Shandong (27.6%) (Ji et al., 2022), northeast China (1.61%) (Zhu et al., 2024), Guangxi (10.7%) (Luo et al., 2023). Our study has revealed a detection rate of 6.10% for BEV within Guangdong Province, which is higher than the rates documented for northeast China. The BEV can be classified into two genotypes, E and F, based on the characteristics of its gene sequence. Genetic evolution analysis of the *5’ UTR* region of BEV reveals that the strains circulating in Guangdong province can be broadly categorized into three clades, encompassing both genotypes E and F. Genotype E exhibits the closest genetic affinity to a strain from buffalo in Guangxi (ON457566.1). One of the populations within Guangdong shows the closest genetic linkage to the Heilongjiang strain (ON986119.1), while another population is most closely related to strains from Henan (MN598018.1) and Shandong (MK639928.1), and is genetically distant from international strains. This suggests that the spread of BEV in Guangdong province is primarily driven by inter-provincial transmission, with genotype F being the predominant strain. All relevant departments are advised to take preventive measures to curtail the spread of these epidemic strains across a wider area.

The study reported that the detection rate of BNoV across China was as follows: northeast China (4.74%) (Zhu et al., 2024), Shandong (9.09%), Sichuan (34.78%), Liaoning (53%), Henan (11.11%), Xinjiang (6.90%) (Wang et al., 2019). Our research findings indicate that only two instances of BNoV were identified in Guangdong province, amounting to a detection rate of 0.68%. Additionally, the prevalence of the virus displayed a marked variation across the various regions within the province. A total of 2 cases of BNoV were detected in Guangdong Province, with a detection rate of 0.68%. Phylogenetic analysis of the *Rdrp* gene of BNoV revealed that the Guangdong strain was closely related to the Hebei strain, both of which belonged to the GIII.2 subgenotype. The GIII.2 subgenotype is also prevalent in many areas of China, and the phylogenetic tree indicated that several domestic strains clustered together. This suggests that the epidemic of BNoV in each province is likely caused by domestic strains rather than imported ones. Therefore, the primary strategies for the prevention and control of BNoV should be directed toward enhancing the quarantine measures for cattle imported from other provinces, thereby mitigating the potential dissemination of BNoV strains across provincial boundaries.

BCoV has an overall detection rate of 30.8% in China, with the detection rate in the South China region as high as 60.5%. Guangxi Province has the highest detection rate at 90% (Geng et al., 2023), while in this study and its neighboring Guangdong Province, the detection rate is only 1.36%, significantly lower than that in Guangxi Province. The phylogenetic tree constructed based on the BCoV *S* gene indicates that the strain under study is most closely related to the Sichuan strains (MK095181.1 and MK095182.1). It also clusters together with the Shaanxi strain (OP866727.1), the Shandong strain (MZ711359.1), and other domestic strains in the same major clade. In contrast, the American classical strain “Mebus” (U00735.2) and other international strains are located in a separate major clade, suggesting a relatively distant genetic relationship. The homology analysis of the *S* gene of BCoV revealed that the *S* gene sequence of the Guangdong BCoV strain exhibits a low degree of similarity to the sequence of the classic American “Mebus” strain, characterized by 36 nucleotide mutations and 28 amino acid substitutions. According to the homology analysis results of BCoV *S* gene, since the clustering of BCoV is determined by the geographical origin of the strains (Abi et al., 2020), it is suspected that the BCoV strains circulating in most areas of China are continuously evolved from local strains, rather than imported strains. The high homology with strains from other domestic regions suggests that this strain is relatively stable and has not undergone major mutations during its spread. Considering the high prevalence of BCoV in China (Geng et al., 2023), Guangdong Province should pay particular attention to the quarantine of items related to the cattle industry that enter and exit from neighboring Guangxi Province, strictly controlling the geographical boundaries to prevent the invasion of wild strains.

The detection rate of BRV in Guangdong Province is 10.85%, lower than the overall national detection rate of 46% (Chen et al., 2022), and slightly lower than that in other regions. The G6 and G10 subtypes are the main BRV strains circulating in China. According to the phylogenetic analysis of the *VP7* gene, all Guangdong strains belonged to the G6 subtype, which is the main epidemic strain in China. Given the characteristic of rotavirus to undergo genetic recombination in different species (Singh et al., 2022; Tsugawa et al., 2021), this strain may have the risk of cross-species transmission. Therefore, the prevention and control of BRV should not only target cattle but also focus on preventing cross-species transmission and the occurrence of genetic recombination. This would make the genes of BRV more complex and difficult to control.

Our study findings reveal that the prevalence of BVDV in Guangdong Province was a modest 2.03%, with the occurrence of BVDV-induced diarrhea in calves being relatively mild. At present, the predominant strains circulating in China are the 1b and 1 m subtypes. According to the genetic evolution analysis of the BVDV *5’UTR* sequence, the strains detected in Guangdong province are closely related to the Beijing strain (KF925512.1) and the Sichaun strain (MG323518.1), which belongs to the BVDV-1b subtype, indicating that this subtype is the dominant strain in China. Since the initial report of BVDV in the United States, the infection prevalence in North and South America has soared to over 50% (Moennig et al., 2005). Conversely, in the Chinese provinces of Hebei, Shandong, and Henan, the seropositivity rates for BVDV neutralizing antibodies stand at 40.8, 83.3, and 53.8%, respectively (Ran et al., 2019). Meanwhile, research has uncovered a shockingly high prevalence of BVDV in Inner Mongolia, China, with the infection rate hitting 88.9% (Gao et al., 2013)— a figure that signals a critical alert. The findings of our current study indicate a relatively low detection rate of Bovine Viral Diarrhea Virus (BVDV) in Guangdong province. Nonetheless, it remains imperative to uphold rigorous and ongoing prevention and control strategies to safeguard against the potential introduction of more virulent BVDV strains into the region.

## 5 Conclusion

In summary, although the proportion of positive samples for BToV, BEV, BNoV, BCoV, BRV, and BVDV was not high in all samples, it is important to note that BToV and BNoV were detected for the first time in Guangdong Province. As new infectious diseases causing diarrhea without vaccines or specific drugs, they pose potential threats to the cattle industry. Therefore, we need to carry out epidemiological investigation all the time by collecting a large number of samples in Guangdong province to facilitate the development of prevention and control programs.

## Data Availability

The data presented in the study are deposited in the NCBI repository, BioProject ID: PRJNA1153982.
